# Climate Change and Apple Farming in Indian Himalayas: A Study of Local Perceptions and Responses

**DOI:** 10.1371/journal.pone.0077976

**Published:** 2013-10-30

**Authors:** Basavaraj Basannagari, Chandra Prakash Kala

**Affiliations:** Ecosystem and Environment Management, Indian Institute of Forest Management, Bhopal, Madhya Pradesh, India; University of Florida, United States of America

## Abstract

Apple farming is an important activity and profession of farmer communities in the Himalayan states of India. At present, the traditional apple farming is under stress due to changes in climate. The present study was undertaken in an Indian Himalayan state, Himachal Pradesh, with the major aim of studying perceptions of farmers on the effects of climate change on apple farming along the altitudinal gradient. Through questionnaire survey, the perceptions of farmers were recorded at low hills (<2500 m), mid-hills (2500–3000 m), and upper hills (>3000 m). At all elevation range the majority of farmers reported that there was increase in atmospheric temperature, and hence at low hills 72% farmers believed that this increase in temperature was responsible for decline in fruit size and so that the quality. Thirty five percent farmers at high hills and 30% at mid hills perceived frost as a major cause for damaging apple farming whereas at low hills 24% farmers perceived hailstorm as the major deterrent for apple farming. The majority of farmers, along the altitude (92% at high hills, 79% at mid hills and 83% at low hills), reported decrease in snowfall. The majority of farmers at low altitude and mid altitude reported decline in apple farming whereas 71% farmers at high hill areas refused decline in apple farming. About 73–83% farmers admitted delay in apple’s harvesting period. At mid hills apple scab and at low hills pest attack on apple crops are considered as the indicators of climate change. The change in land use practices was attributed to climate change and in many areas the land under apple farming was replaced for production of coarse grains, seasonal vegetables and other horticulture species. Scientific investigation claiming changes in Indian Himalayan climate corroborates perceptions of farmers, as examined during the present study.

## Introduction

The mountain ecosystem is one of the most vulnerable ecosystems to the climate change and so that the mountain communities, especially those mainly depend on animal husbandry, marginal agriculture and horticulture products. The Himalayan mountain ecosystem, at present, is facing the challenges created due to increasing aridity, warmer winter season, variability in precipitation, and unexpected frosts and storms [1], [2], which largely affect the entire range of biodiversity, including agriculture and horticulture crops [1], [3]. Though, the Himalaya harbours rich biodiversity and is one of the most vulnerable mountain ecosystems to climate change [4], [5], there is paucity of systematic analysis of climate change and its impacts on the Himalayan ecosystems, biodiversity and local people’s livelihoods [6].

Farmers of Indian Himalayan region grow many fruit crops, including pomes (apple and pear) and stone fruits (peach, plum, apricot and cherry) in considerable quantity [7]; however apple has the preference over all other horticultural crops [8]. Worldwide, there are over 7,500 known cultivars of apples [9]. China dominates the world in production of apple, followed by the United States, and India ranks seventh with average yield of about 7.24 tonnes per hectare [9]. All three north-west Himalayan states of India - Himachal Pradesh, Jammu-Kashmir, and Uttarakhand are the major apple producing states of India. In these states, the apples are grown at altitude ranging from 1200 m to 3500 m above mean sea level [10].

In Himachal Pradesh, the area under apple has increased from 400 ha in 1950–51 to 3,025 ha in 1960–61 and further 99,564 ha in 2009–10 [11], [12]. Though the production of apple in this state has steadily increased by bringing more areas into apple farming, the productivity has declined [13]. The present study, therefore, aims to understand the causes of reducing apple farming in the state despite high preference of local people to continue apple farming. Apple being highly sensitive to adversities of climate [14]–[16], the centre of attention of this study is to access the perceptions of farmers on effects of climate change on the apple farming along the altitudinal gradient.

## Methods

### Study Area

The present study was carried out in the Kinnaur district of Himachal Pradesh, which is located in the northern India. Himachal Pradesh is bordered by Jammu-Kashmir on north, Punjab on west and south-west, Haryana on south, Uttarakhand on south-east and China on the east [12]. The state is second largest producer of apples in the country. About 66% of area in the state is under forest cover, which is rich in biodiversity including medicinal and aromatic plants. The main occupation of the people of this state is agriculture, horticulture and allied sector. The topographical variations and altitudinal differences provide congenial environment for cultivation of temperate to sub-tropical fruits.

Intensive investigations at village level were carried out in the Kinnaur district of Himachal Pradesh. Kinnaur district is situated on both sides of river Satluj from 31°-05′-50″ to 32°-05′-15″ north latitude and between 77°-45′ to 79°-00′-35″ east longitudes. Of the 12 districts of Himachal Pradesh, Kinnuar being third largest district spans over 6,520 km^2^. Total human population of this district is 84,298 and the population density is estimated as 15 per km^2^ [17]. It is situated between 2,100 m to 3,600 m above mean sea level, and it is popularly known as the ‘apple bowl’ of the state. Kinnaur district is composed of 3 administrative blocks such as Nichar, Kalpa and Pooh.

### Field Surveys

The field surveys were undertaken in 2012. The approval of ethical committee was not required as the present study does not deal with the clinical trials on humans and animals. The study rather deals with the agro-biodiversity, which falls in the jurisdiction of Biological Diversity Act 2002. The regulations under ‘Assess to Biological Diversity’, Chapter 2, Section 3.0 and Section 4.0, authorize authors, being the Indian citizens, to carry out research and publish research papers with respect to agro-biodiversity in India. The present study complies with all relevant regulations.

Random survey was carried out in all three development blocks of Kinnuar district - Kalpa, Pooh and Nichar along the altitudinal gradient (e.g., upper hills, mid-hills, and low hills). Two villages at each altitudinal range (e.g., upper hills >3000 m; mid-hills between 2500–3000 m; low hills <2500 m) were selected. Villages Pooh and Chango were sampled at the high hills (>3000 m), Kalpa and Sangla at the mid hills (2500 m–3000 m) and Nichar and Urni were selected for sampling at the low hills (<2500 m). A total of 300 respondents inhabited in 6 villages were interviewed during the present investigations along the altitudinal gradient in Kinnaur district.

The data were obtained with the help of structured questionnaire survey. Before conducting the survey, we informed and discussed with the participants about the nature of the research. The subject’s participation was voluntary. Fifty households in each village were randomly sampled and information was gathered on various parameters, including perceptions of the farmers on the effects of temperature and precipitation on apple farming. Besides, Participatory Rural Appraisal was also used for collection of data through interviews/discussions with individual and focus groups within the farmer’s community. Farmers were also interviewed on the trends in snowfall, extreme events, and changes in seasonality, cropping system and pest attack.

### Secondary Information

An extensive literature survey was carried out on the parameters related to research study. Different Departments (e.g., State Forest Department, State Horticulture Department, State Universities and other Government Departments) were approached for collection of present and past information related to apple farming in the state of Himachal Pradesh.

## Results

The horticulture has emerged as the main profession of inhabitants in the study area, followed by agriculture and animal husbandry. Of the total people interviewed, 73% admitted horticulture as a primary occupation, followed by agriculture (23%). The majority of farmers (78%) at low altitude (<2500 m) and 72% at mid altitude (2500–3000 m) reported in decline of apple farming whereas majority of farmers (71%) of high hill areas (>3000 m) admitted that there was no decline in the apple farming.

**Figure 1:**
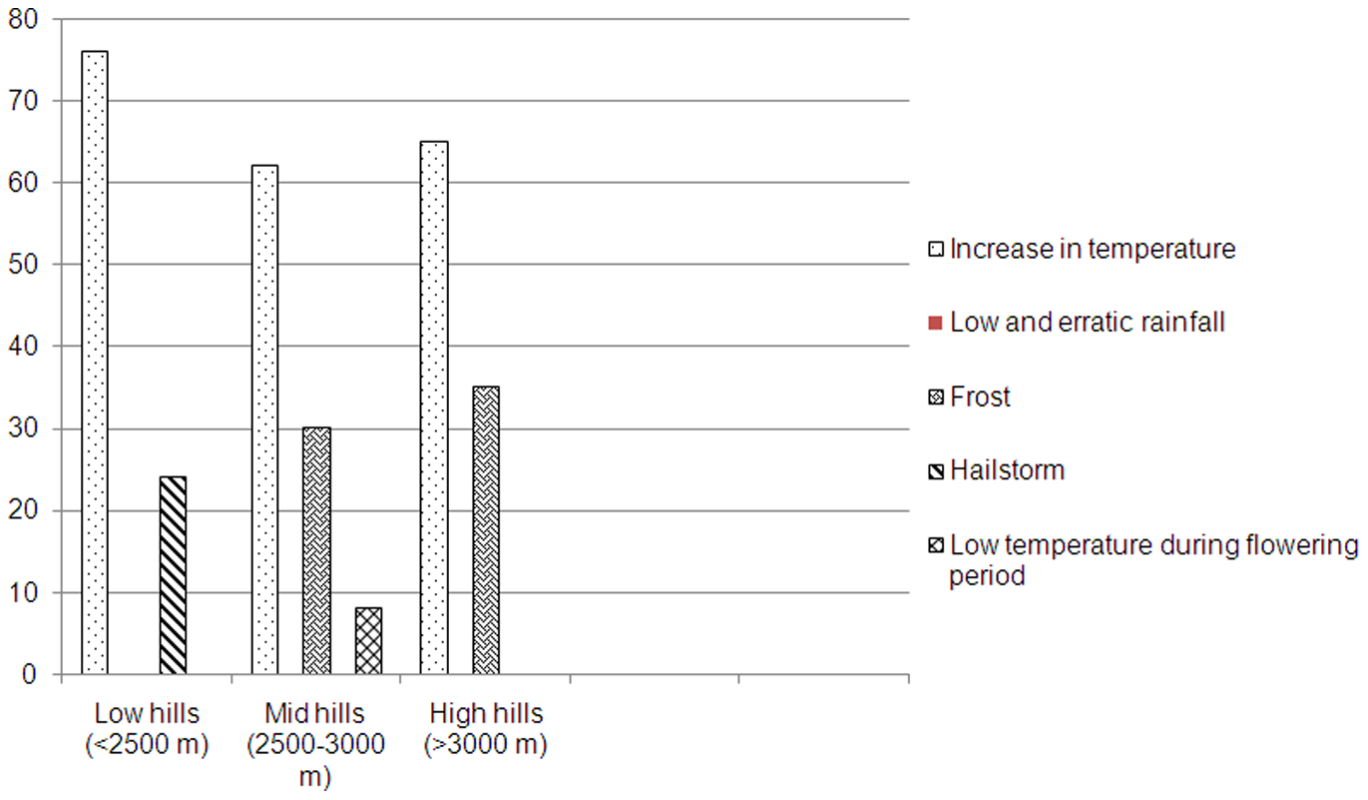
Perceptions of respondents on the various factors responsible for damage of apple farming along the altitudinal gradient in the Kinnaur district of Himachal Pradesh.

**Figure 2:**
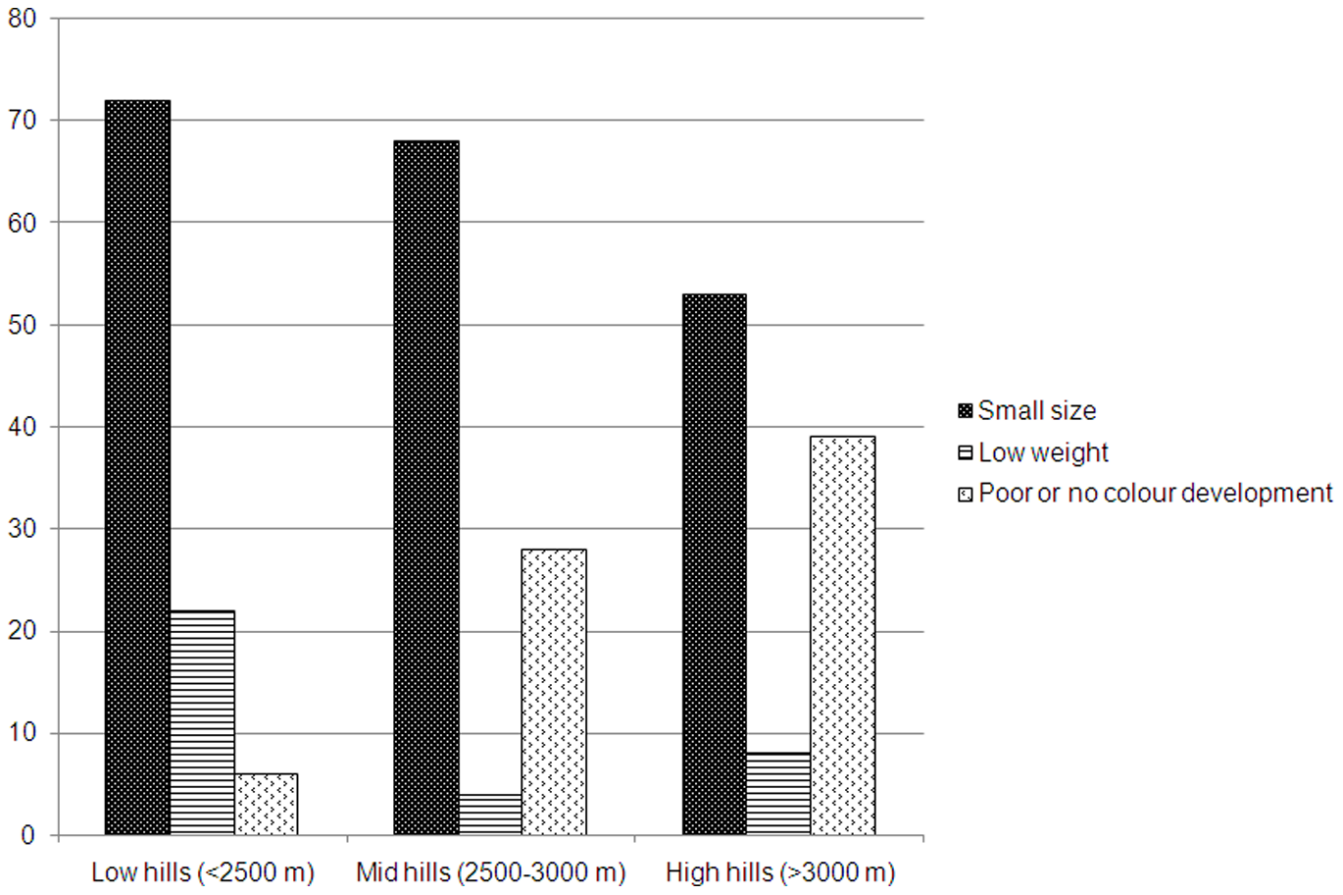
Perceptions of respondents along the altitudinal gradient in the Kinnaur district of Himachal Pradesh on the impact of fruit quality due to climate change.

Along the altitudinal gradient the majority of respondents reported that there was increase in atmospheric temperature. About 24% of farmers at low hills perceived hailstorm as the major deterrent for apple farming whereas 35% farmers at high hills and 30% at mid hills perceived frost as a major cause for damaging apple farming (**Figure 1** **)**. The perceptions of growers on the quality of apple fruit production varied along the altitudinal gradient. About 72% farmers at low hills believed that change in climate, especially increasing temperature, was responsible for decline in fruit size and so that the quality. Lacking of appropriate fruit colour, due to climate change, was considered as deterrent factor in maintaining the fruit quality by 39% respondents at high hills **(** **Figure 2** **)**.

**Figure 3:**
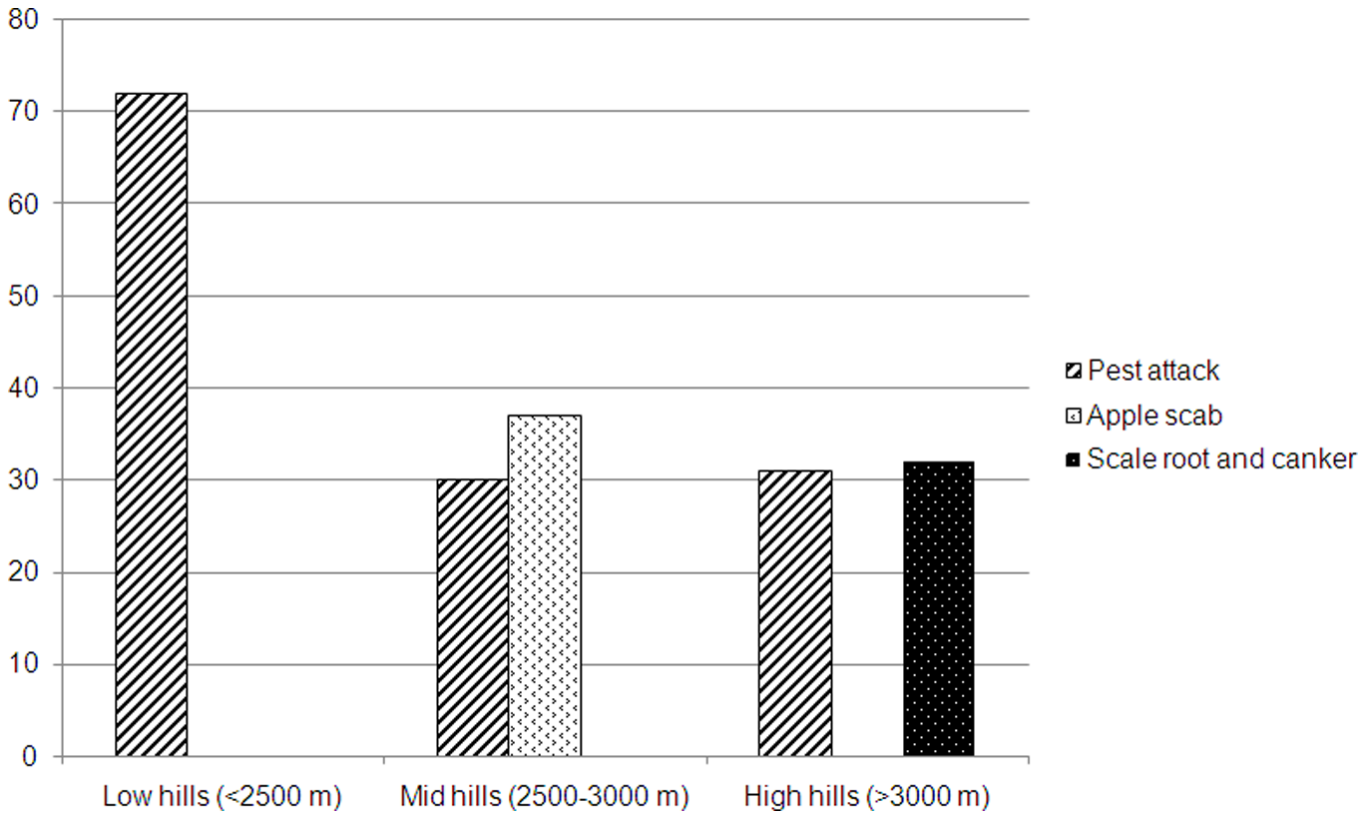
Indicators of climate change impacts on the apple crop as per the perceptions of respondents along the altitudinal gradient in the Kinnaur district of Himachal Pradesh.

**Figure 4:**
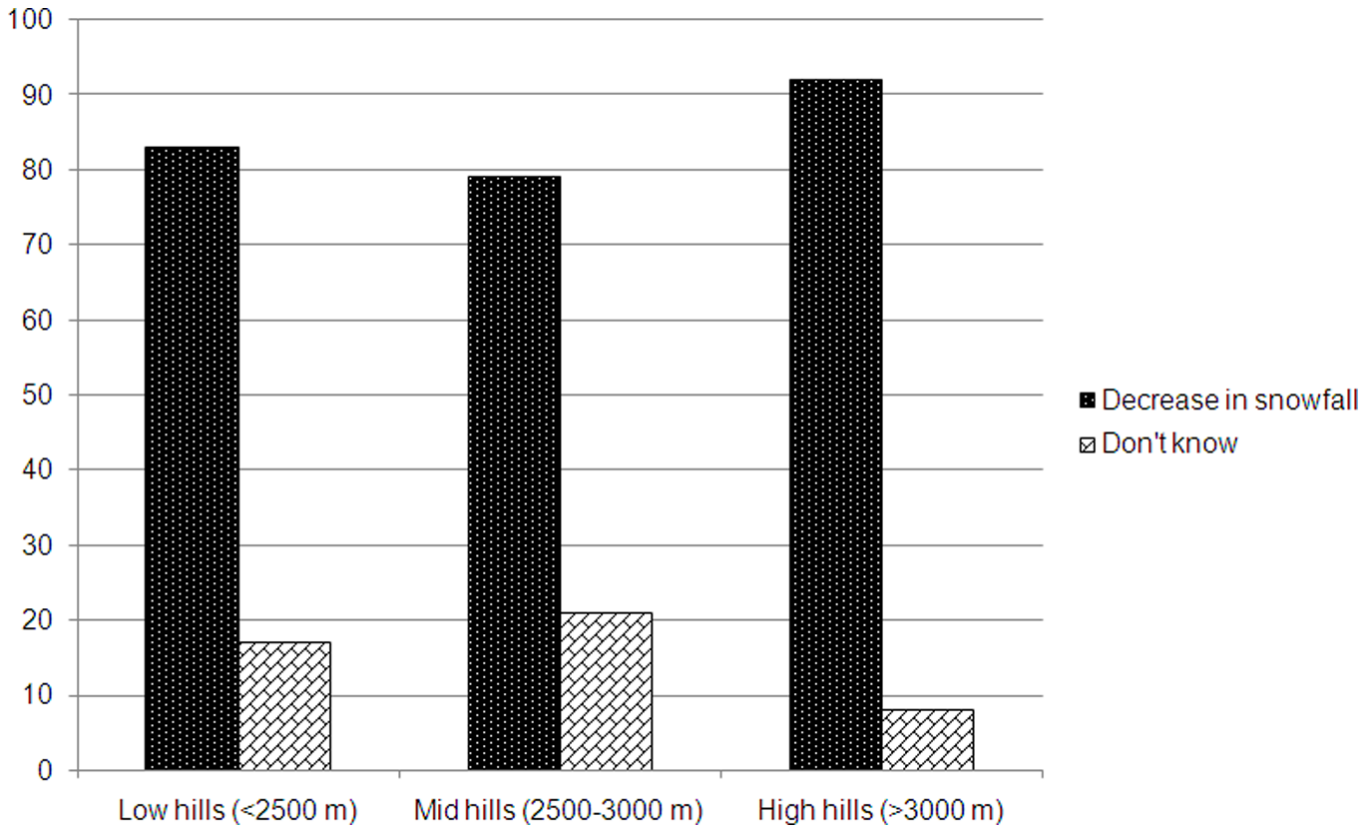
Perceptions of respondents along the altitudinal gradient in the Kinnaur district of Himachal Pradesh on the snowfall pattern.

The growers reported many indicators of climate change that impact apple farming along the altitudinal gradient. Infestation of pest and diseases such as apple scab, scale root and canker were some the indicators of climate change that increased the cost of production due to increase in use of pesticides and chemical fertilizers. At low hills majority of respondents reported pest attack on apple crops as one of the indicators of climate change whereas at mid hills apple scab was the most prominent (**Figure 3**). Apple production is known to influence by chilling hours however, the majority of respondent along the altitude (92% respondents at high hills, 79% at mid hills, and 83% at low hills) reported decrease in snowfall. None of the respondents in the study villages admitted that there was increase in snowfall **(** **Figure 4** **)**.

**Figure 5:**
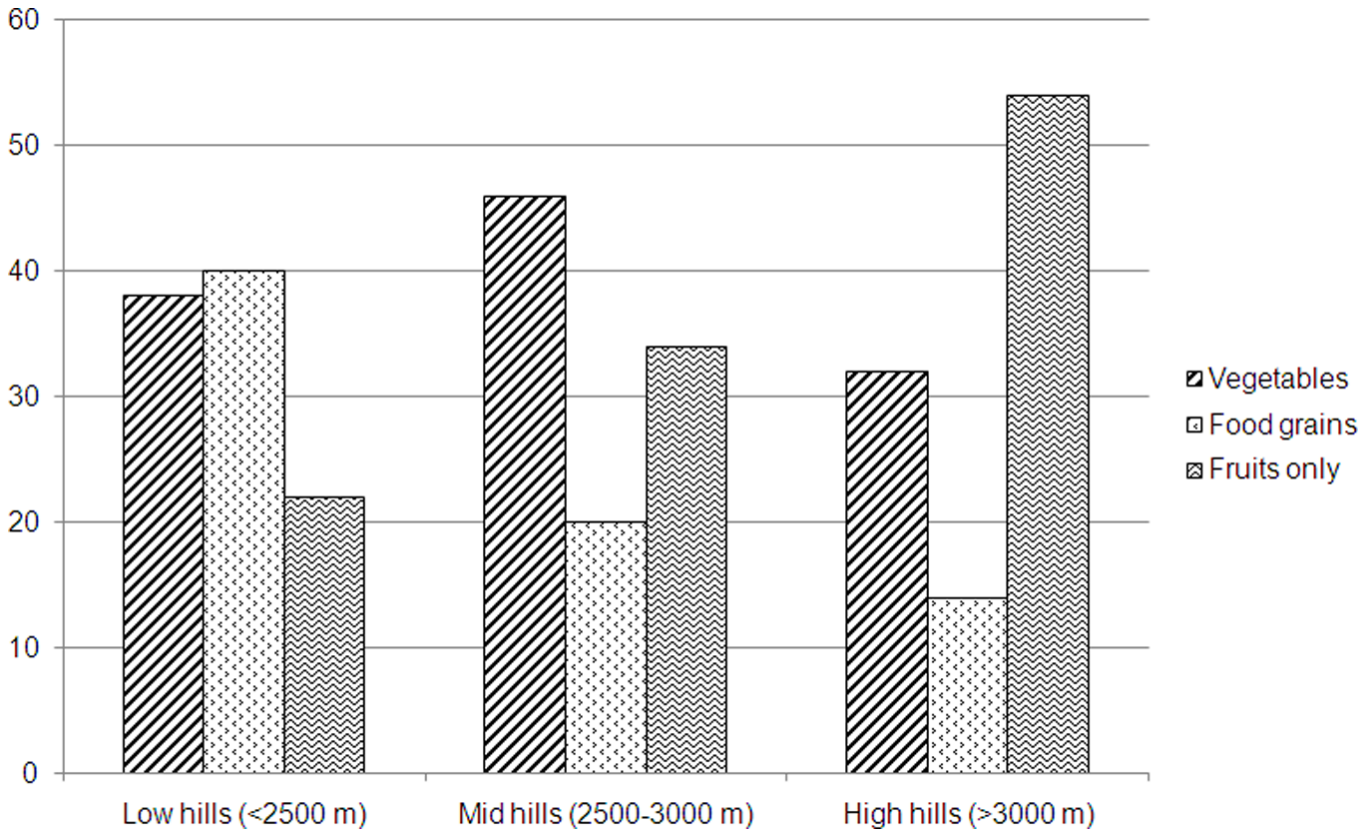
Conversion of apple farming into other land-use practices including cereals and vegetables along the altitudinal gradient in the Kinnaur district of Himachal Pradesh.

**Figure 6:**
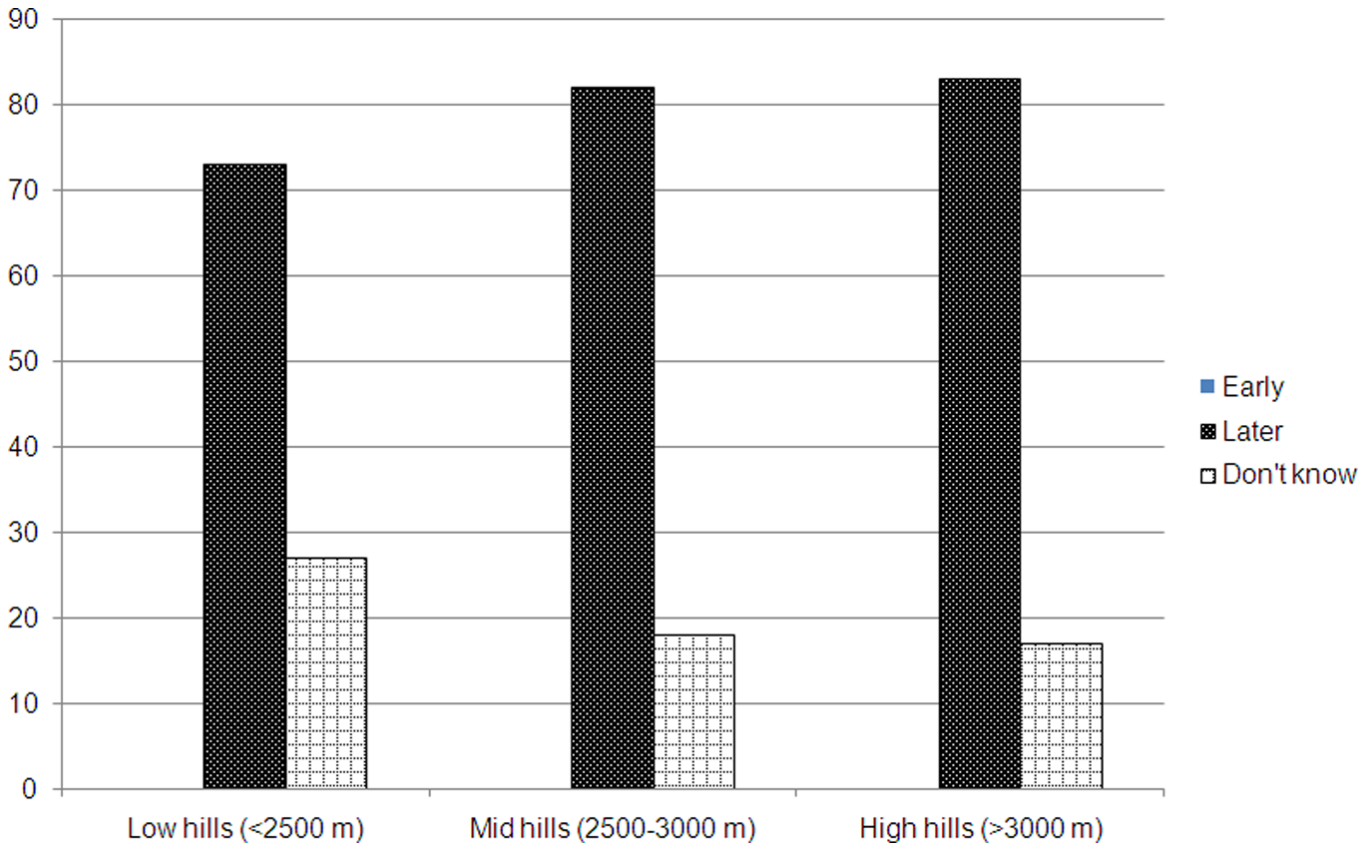
Effects of climate change on the harvesting period of apple along the altitudinal gradient in the Kinnaur district of Himachal Pradesh.

The growers indicated that because of climate change the land-use practices were under change. In many areas the land under apple farming was replaced by farming of coarse grains, seasonal vegetables and other horticulture species (**Figure 5** **)**. The majority of respondents reported that there was shift in harvesting period of apple due to climate change, especially of increasing temperature, across the altitudinal gradient (**Figure 6** **)**. Seventy three to eighty three percent respondents admitted that apple harvesting period has been delayed in the study villages.

## Discussion

The area brought under apple farming in Himachal Pradesh has increased from 35,076 ha to 99,564 ha during 1975–2010 however, the same time series data show erratic trend in annual apple production, which is low and not in the proportion of land brought under apple farming **(** **Table 1** **)**. The erratic trend in apple production may have number of reasons, including farming sites on sloppy and marginal lands, areas prone to adverse weather conditions, rainfed land and small land holdings [18], [19]. Nurseries are being mainly developed by utilizing scion wood of old varieties and seedling rootstock for propagation. The concept of budwood bank for supply of certified quality true-to-type planting material for further propagation is lacking [18]. Apart from this, climate change impacts the apple farming in numerous ways.

**Table 1:** Production of apple in Himachal Pradesh; Source: [18], [36].

Year	Production (mt)	Area (ha)
1975–76	200000	35076
1981–82	306798	45335
1989–90	394868	59988
1990–91	342071	62828
1991–92	301730	66767
1992–93	279051	69429
1993–94	294734	72406
1994–95	122782	75469
1995–96	276681	78292
1996–97	288538	80338
1997–98	234253	83056
1998–99	393653	85631
1999–2000	53000	88631
2000–01	376736	90347
2002–03	348263	92820
2003–04	459492	84112
2004–05	527601	86202
2005–06	540356	88560
2006–07	268402	91804
2007–08	592576	94726
2008–09	510161	97438
2009–10	280105	99564

Decline in atmospheric moisture and increase in frequent droughts may lead to higher temperature as reported by farmers along the altitudinal gradient during the present course of investigations. The winter temperature and precipitation in the form of snow are very important and sensitive climatic factors for induction of dormancy, bud break and also to ensure proper flowering in apples [20]. Apple requires 1200–1500 hours of chilling depending on type of cultivars. The chilling hours <1000 lead to poor fruit formation [21]–[23]. Prolonged delay in cold in December and January severely affects the chilling requirements [24]. Apple grows best in the regions where the tree undergoes an uninterrupted winter rest [25].

However, the extreme minimum temperature during the winter causes winter freeze injury in apple fruits, which results poor apple yield [26]. Summer temperature and climate conditions also influence the size and quality of apples as the fruits develop during April to June. The high (>26°C) or low temperature (≤15°C) during flowering phase reduce apple crop [27]. Temperature impacts apples farming throughout the season from immediately after blooming period in the apple orchards to the fruit size at harvest [28]. Delicious group of apple trees, the one which is mostly cultivated in the present study area, under normal conditions require 1234 units of winter chilling [29]. However, some of the low altitude zones under apple cultivation do not fulfil sufficient winter chilling due to rise in temperature. With deficiency in chilling hours, flower buds produce fewer fruit clusters resulting in delay in bloom period.

The increasing pattern of chill unit at 2700 m above mean sea level reports that the area is conducive for apple cultivation and hence there is a shift in apple farming from low hills to middle and high hills [15], [16]. These reports support the farmer’s perceptions of the present study area which showed that apple cultivation shifts from low to high elevations with respect to increase in temperature. The harvesting period of apple is also delayed for a week to a fortnight. Apart from temperature, the decline in apple farming at low hills is also attributed to hailstorms, decrease in snowfall and inferior quality of fruit production due to pest attack. In many such areas, the apple farming is being replaced by raising course grains and seasonal vegetables.

Our results with respect to the effects of climate change on apple farming are similar to those inferred for other regions of the world. In Japan, the areas suitable for apple farming (i.e. with an annual average temperature of 7∼13°C) are gradually shifting northward besides decline in colour quality of apple due to heat injury [30], [31]. Farmers, here, have also started shifting from apple to peach farming because peach farming is considered less vulnerable to climatic stresses [32]. The apple farming areas have shifted north and or to highlands in the Yangu of Gangwon Province from Daegu of Gyeongbook Province of Korea, as well [31]. The decrease in number of chilling hours in four mountain oases of Oman has made to decline the production of apple, and since such fruit trees barely fulfil their chilling requirements such marginal fruit production is expected to decline further [33]. In the neighbouring country of India, about 90% farmers believe that climate change is the major factor responsible for decline in apple production [34].

A model demonstrating climatically suitable areas for growing apples suggests that as temperature increases the areas suitable for growing apples move from south to north, from coast to midland, from planes to mountains and from urban centre to suburban areas [35]. The Indian Himalaya has warmed by 1.5°C from 1982 to 2006, at an average rate of 0.06°C yr ^−1^, which is considerably higher than the global average [6]. Majority of farmers during the present study believe that there is change in climate, especially increase in atmospheric temperature and decrease in snowfall, which they consider most responsible for decline in fruit size and so that the quality of apples. Scientific investigation claiming that the Indian Himalaya has warmed by 1.5°C from 1982 to 2006 corroborates such beliefs of farmers, as examined during the present study. The increase in temperature throughout the Himalaya makes it likely that our findings with respect to climate change effects on apple farming may represent a trend across the Himalaya. It is assumed that other fruit production regions, where similar sensitive fruit crop species are grown under the climatic conditions similar to the Himalaya, are also susceptible to even slight warming.

Apple is known in Himachal Pradesh as a most significant commercial fruit crop. However, in the study area, the farmers are mainly customized to follow the traditional and age-old practices of cultivation. They are less aware about scientific agro-commercial practices, horticulture schemes and agri-inputs due to lack of communication facilities at high hills. The present changes in climatic conditions such as change in temperature, precipitation, ground frost and hailstorm, and subsequent adversities in terms of proliferation of insect-pest and diseases, loss of soil fertility, water availability, and natural calamities pose serious threats on apple production. Apart from climate change, the apple production may be declined due to continuation of plantations that have crossed their fruit bearing stage. There is a need for re-plantation of apple trees in a systematic manner on a regular basis. Besides, it is important to understand the variations in the patterns of climate change and also to identify management practices and alternatives for farmers in order to cope up the vagaries of changing climate.
